# Editorial: Neuroimaging for the measurement and management of pain

**DOI:** 10.3389/fneur.2022.1029238

**Published:** 2022-09-28

**Authors:** Marie-Eve Hoeppli, Susanne Becker, Marina López-Solà, Flavia Di Pietro

**Affiliations:** ^1^Pediatric Pain Research Center (PPRC), Cincinnati Children's Hospital Medical Center, Cincinnati, OH, United States; ^2^Division of Behavioral Medicine and Clinical Psychology, Cincinnati Children's Hospital Medical Center, Cincinnati, OH, United States; ^3^Department of Chiropractic Medicine, Balgrist University Hospital, University of Zurich, Zurich, Switzerland; ^4^Clinical Psychology, Department of Experimental Psychology, Heinrich Heine University, Düsseldorf, Germany; ^5^Department of Medicine, School of Medicine and Health Sciences, University of Barcelona, Barcelona, Spain; ^6^Curtin Medical School, Curtin University, Bentley, WA, Australia; ^7^Curtin Health Innovation Research Institute, Curtin University, Bentley, WA, Australia

**Keywords:** pain, brain imaging, chronic pain, biomarkers, resting state functional magnetic resonance imaging (fMRI), voxel-based morphometry (VBM)

The article collection titled *Neuroimaging for the Measurement and Management of Pain*, a Research Topic within Frontiers in Neurology: Applied Neuroimaging, sought to bring together quality neuroimaging studies of the role of the central nervous system in pain. Pain is enormously complex. While there has been great progress in neuroimaging technology and methodology there is still much we do not understand about the nervous system in pain disorders, particularly the role of the spinal cord, and there is ongoing debate around optimal practices for data analysis and interpretation.

The Research Topic includes work from 59 authors across 7 countries, and a range of issues is addressed. New and exciting directions in data analysis and machine learning are presented, as well as neuroimaging investigation of the spinal cord. An important inclusion is the exploration of the fundamental social and emotional influences of pain, and structural neuroimaging work on specific pain disorders—both somatic and visceral. Overall the collection of rigorous work showcases the progress made in this field and the bright future ahead.

## Exciting directions in machine learning and the development of biomarkers

Lamichhane et al. used graph theory measures derived from resting state functional connectivity (FC) scans to achieve high classification accuracy of low back pain patients from healthy controls. They raise the question of whether graph matrices from resting state functional magnetic resonance imaging (rsfMRI) might be useful as brain biomarkers of low back pain, particularly when supplemented by a hybrid feature selection method to remove redundant variables thus improving machine learning classification accuracy.

In their review, Zhang et al. discuss the costs and benefits of brain biomarkers for chronic pain. Even with the advent of advanced analysis such as machine learning, there remains a significant knowledge gap—how does nociception result in pain? Thus the authors focus on the concept of learning in the emergence of pain and the limbic brain circuitry and dopaminergic signaling. Together with the use of big data, machine learning and the use of hybrid feature selection models, they propose that biomarker learning is possible and has potential for clinical translation.

Finally, in a perspective piece that calls to incorporate the biopsychosocial approach to neuroimaging biomarker development, Reddan argues that three levels of inquiry should be addressed to increase the clinical relevance of pain neuroimaging models. Needed first is more diverse sampling for the development of diagnostic biomarkers (population-based, nomothetic approach); second is the development of treatment-relevant models tailored at the individual level (person-based, idiographic approach); and third is prevention-relevant models that combine neuroimaging data and one's own socioeconomic conditions (social epidemiologic approach). The author recommends ways that pain's complexity can be leveraged in service of the individual and society.

## Personalizing treatment and predicting the transition to chronicity

Although not directly measuring pain as an outcome, Su et al. explored the potential of preoperative brain biomarkers for prediction of recovery from cervical spondylotic myelopathy (CSM). Using rsfMRI and machine learning, the authors were able to classify CSM patients from healthy controls with high classification accuracy, and furthermore predict neurological recovery in CSM patients. Cross-site validation analyses demonstrated good reproducibility and generalization, and thus the study provides a step toward novel strategies of predicting neurological recovery—with clear implications for the use of such techniques and analyses in chronic pain.

Also using rsfMRI to determine the predictive value of brain networks, Danyluk et al. recruited a sample of trigeminal neuralgia patients. They compared FC between limbic and accessory sensory networks in patients and healthy controls, as well as determining whether pre-operative variability in such networks might distinguish responders from non-responders to surgery. Their results suggest not only FC differences in the TN patients, but differences in FC in the limbic system between those patients who did and did not respond to surgery. They also present interesting correlations between brain FC and illness duration.

In their review, Kandic et al. sought to elucidate the brain circuits implicated in pain chronicity. They review the evidence from non-invasive brain stimulation studies, focussing on the motor cortex and the dorsolateral prefrontal cortex, to explore the transition from acute to chronic pain. The evidence presented also provides exciting potential targets for non-invasive stimulation as therapy, in the transition to the chronic phase.

## Investigation and stimulation of the spinal cord

Owing to exciting developments in technology and methodology, MRI of the spinal cord is now possible and increasingly available. Martucci et al. conducted an rsfMRI investigation of the spinal cord in fibromyalgia patients both taking and not taking opioid medications, as well as pain-free controls. Interestingly, regional spinal cord activity in the opioid group was more similar to controls, whereas the non-opioid group displayed differences in both ventral and dorsal spinal cord activity (low frequency fluctuations). In further exploration, fatigue was found to be correlated with regional spinal cord activity differences.

In an aim to explore the efficacy of different settings (tonic and burst) of implanted spinal-cord stimulation in chronic pain, Niso et al. assessed the influence of attention on the somatosensory-evoked brain responses (SEPs) read with electroencephalography (EEG). Late SEP responses were reduced in both the attended and unattended (mind-wandering) conditions in the burst stimulation group, but only in the unattended condition in the more traditional tonic stimulation group. They propose that neuroimaging could potentially be used to personalize spinal-cord stimulation treatment in chronic pain.

## Emotional and social influences on pain

Lyu et al. explored the modulation of pain by emotion. By recording the EEG response to an electrical painful stimulation primed by visual images of different emotional valence (negative, positive and neutral), the authors were able to demonstrate that pain unpleasantness, but not pain intensity, was modulated by emotion. Further, they report two gamma band oscillations (GBO), one early and one late, differentially related to pain intensity and unpleasantness. They conclude that the early GBO might reflect the sensory dimension of pain, while the affective dimension might be related to the late GBO component.

Sharvit and Schweinhardt review the effects of social manipulation on pain, highlighting that the social influences of pain have been under-studied in neuroimaging, in contrast to the psychological. Indeed the authors remind us of the inclusion of the social dimension in a recently updated definition of pain from the International Association for the Study of Pain. Discussing social manipulations such as verbal and non-verbal signals and social support, the authors present a schematic summary graphic of different social modulatory themes on pain intensity ratings.

## Structural brain alterations in specific chronic pain states

Domin et al. investigated gray matter volume alterations in the brains of upper limb complex regional pain syndrome (CRPS) sufferers, as well as their association to clinical characteristics and sensorimotor performance. CRPS patients showed lower GMV in the bilateral thalamus; there were associations with pain intensity and duration in the ACC, and associations between the posterior insula and sensorimotor performance. Also investigating limb pain using MRI, Wei et al. studied the structural asymmetry of the pre and post central gyrus in chronic shoulder pain. They report precentral gyrus surface area asymmetry, between pain (according to pain side) and pain-free controls. Further, fMRI and seed-based FC analysis showed significant group differences in the postcentral gyrus and other areas. Notably, both structural and functional imaging asymmetry was correlated with pain and functional impairments.

By no means as widely studied as chronic musculoskeletal pain, Ohlmann et al. report on two cohorts of chronic visceral pain: ulcerative colitis and irritable bowel syndrome (IBS), as well as a comparative healthy control group. GMV was reduced in the frontal cortex and the anterior insula in ulcerative colitis. In IBS there were more widespread differences comprising both increases and decreases in GMV in several brain areas and networks. Interestingly, there was an association between visceral symptoms and GMV in frontal brain regions in both groups. Future work will hopefully elucidate the partly distinct alterations in brain morphology in patients with chronic inflammatory vs. functional bowel disorders.

Together, the studies in the Research Topic, *Neuroimaging for the Measurement and Management of Pain*, address fundamental aspects of pain and its complexity, and will contribute to push the field forward with explorations of the spinal cord and with work on the potential for brain biomarkers of pain, its prognosis and its response to therapy.

## Author contributions

All authors listed have made a substantial, direct, and intellectual contribution to the work and approved it for publication.

## Funding

ML-S is supported by the Serra Hunter Programme of the Generalitat de Catalonia, Spain.

## Conflict of interest

The authors declare that the research was conducted in the absence of any commercial or financial relationships that could be construed as a potential conflict of interest.

## Publisher's note

All claims expressed in this article are solely those of the authors and do not necessarily represent those of their affiliated organizations, or those of the publisher, the editors and the reviewers. Any product that may be evaluated in this article, or claim that may be made by its manufacturer, is not guaranteed or endorsed by the publisher.

